# Editorial: Sudden Death in Epilepsy: Basic and Translational Research

**DOI:** 10.3389/fneur.2018.00484

**Published:** 2018-06-27

**Authors:** Christopher M. DeGiorgio

**Affiliations:** UCLA Department of Neurology, Olive View-UCLA Medical Center, David Geffen-UCLA School of Medicine, Los Angeles, CA, United States

**Keywords:** sudden unexpected death in epilepsy, epilepsy, seizures, drug resistant epilepsy

Sudden Unexpected Death in Epilepsy (SUDEP) is an important cause of death in people with epilepsy, especially those with chronic epilepsy. In a UK cohort, SUDEP accounted for 17% of deaths in those with chronic epilepsy (1). The incidence of SUDEP is 0.2 per 1,000 persons per year in children, and 1.0 per 1,000 persons per year inadults (2). The incidence of SUDEP in chronic epilepsy is substantially higher, ranging from 2.64 to as high as 5.95 per 1,000 persons per year (1, 3). In this book genetics, functional imaging, autonomic physiology and clinical risk factors for SUDEP are explored in depth.

## Genetics

Bagnall et al. provide an elegant and comprehensive review of current knowledge of the genetics and basic mechanisms of SUDEP. Several candidate genes are associated with the risk for SUDEP, especially genes associated with long QT interval and cardiac arrhythmias. These include mutations in potassium channels, specifically KCNQ1 and KCNH2 (see Table 2, Bagnall et al.). Other channelopathies associated with SUDEP are caused by defects in sodium channels, especially SCN1A, SCN2A, SCN5A, and SCN8A (Bagnall et al.). Dravet's syndrome, a form of catastrophic childhood onset epilepsy, is associated with the highest recorded SUDEP incidence (9.23 per 1,000 person years) (Bagnall et al.). Over 80% of Dravet's patients express a defect in a subunit of the voltage gated sodium channel SCN1A (Bagnall et al.). Ten percent of SUDEP cases studied expressed a missense or nonsense variant in the gene DEPDC5 (Bagnall et al.). DEPDC5 encodes the protein Egl-10, which regulates the target of rapamycin complex I (mTORC1) (Bagnall et al.). Mutations in DEPDC5 may impart a higher risk of SUDEP, and cause an increase in mTORC1 activity, which is associated with focal cortical dysplasia (Bagnall et al.).

## Clinical risk factors

Since the 1990's, researchers have identified risk factors which are associated with an increase in risk for SUDEP. (3) “Ranking the Leading Risk Factors for Sudden Unexpected Death in Epilepsy” systematically analyzes the major SUDEP risk factors published to date (DeGiorgio et al.). The top 10 risk factors from multiple published cohort studies are ranked using the weighted log of the adjusted Odds Ratio [adjusted log OR/Standard Error], which adjusts the Odds ratio for the size of each study and the confidence interval (DeGiorgio et al.). The top 10 risk factors ranked by the weighted log of the adjusted Odds Ratio are, in order: 1: > 3 GTC seizures per year; 2: > 13 seizures in the last year; 3: No AED treatment in a patient with at least one seizure in the last year 4: > 3 concomitant AED's; 5: > 3 GTCs in the past year; 6: 11–20 GTC seizures in the last 3-months; 7: onset of seizures between birth and 15 years old; 8: IQ < 70; 9: 3 to 5 AED changes in the last year; 10: > 3 concomitant AEDs (DeGiorgio et al.). Note that three or more seizures per year and three or more AED's appear twice in the list, indicating that these factors were validated in more than one study cohort (DeGiorgio et al.). Table 1 by DeGiorgio et al. provides detailed information about each of the top 10 leading risk factors associated with SUDEP.

In another chapter, Dlouhy et al. report a case of SUDEP in a 21-month old little girl who suffered febrile seizures. This tragic case should cause us to take a closer look at any association between febrile seizures and SUDEP, and should remind all SUDEP researchers that behind every SUDEP case reported, there is a human person and a family who suffers (Dlouhy et al.).

## Abnormal autonomic and cardiac physiology

Hampel et al. and Novak et al. explore abnormalities of autonomic function in patients at risk for SUDEP. Hampel et al. examined baroreflex reflex sensitivity, which simultaneously measures beat-to-beat heart rate and blood pressure. Impaired baroreflex sensitivity is often seen in myocardial infarction and heart failure, and is associated with a significantly higher risk of sudden cardiac death (Hampel et al.). High baroreflex sensitivity, expressed as ms/mmHg, is associated with reduced risk for cardiac arrhythmias, and lower baroreflex sensitivity is associated with higher risk (Hampel et al.). Hampel et al. measured baroreflex sensitivity before, during and after seizures in 26 patients with chronic epilepsy hospitalized for epilepsy video telemetry (Hampel et al.). Immediately after seizures, in the post ictal period, baroreflex sensitivity decreased 79% from a pre-ictal value of 15.0–3.1 ms/mmHg, *p* < 0.0001). This important discovery may help explain why most cases of SUDEP are observed in the postictal period, when hypoxia coupled with reduced baroreflex sensitivity increases the risk for lethal cardiac arrhythmias [Hampel et al., (4)].

Novak et al correlated SUDEP risk, as estimated by the SUDEP-7 inventory, with RMSSD, a measure of high-frequency vagus-mediated heart rate variability (Novak et al.). They found that RMSSD is inversely correlated with scores on the SUDEP-7 risk inventory (Pearson *r* = −0.43, *p* = 0.035, Novak et al. Figure 3) (Novak et al.). Subjects with the highest SUDEP-7 scores (higher risk for SUDEP, SUDEP-7 scores between 5 and 7) had significantly lower RMSSD values than subjects with low SUDEP-7 scores (scores < 1). RMSSD values for those with the highest SUDEP-7 scores averaged 17.6 msec SD 5.1, while RMSSD values for the those with the lowest SUDEP-7 scores averaged 32 msec SD 12.5, *p* = 0.03, trend test (Novak et al.). Interestingly, RMSSD values in those at highest risk for SUDEP are similar to values recorded in patients with heart failure, which is consistent with Hampel et al's findings of reduced baroreflex sensitivity following seizures (Dlouhy et al. and Novak et al.). Together, the articles by Hampel at al. and Novak et al. provide evidence that patients at risk for SUDEP have abnormal autonomic function (Dlouhy et al. and Novak et al.).

Polytherapy has been implicated as a risk factor for SUDEP, but polytherapy as an independent risk factor is controversial (5). In an *in-vivo* study, Hulbert et al. found that simultaneous exposure of multiple antiepileptic drugs (carbamazepine, lamotrigine, and levetiracetam) impaired electromechanical coupling in cardiac myocytes. Impaired electromechanical coupling in cardiac myocytes may lead to cardiac arrhythmias (Novak et al.). This finding should lead to a closer evaluation of the risk of multiple AED's in people at risk for SUDEP.

## Functional MRI and functional connectivity

In an elegant functional MRI study (fMRI), Allen et al explored which autonomic structures and networks are abnormal in 32 patients with chronic epilepsy (Allen et al.). Subjects were stratified for risk of SUDEP by age of onset, duration, frequency of generalized tonic–clonic seizures, and nocturnal seizures (Allen et al.). Fourteen subjects were classified as high risk, and 18 were classified as low risk. Those subjects at high risk for SUDEP demonstrated significantly reduced functional connectivity in the thalamus, midbrain, anterior cingulate, putamen and amygdala (Allen et al.). This report provides key evidence of an anatomic and functional defect in key structures which regular sympathetic and parasympathetic activity in patients at risk for SUDEP (Allen et al.).

## Inflammation

Inflammation is believed to contribute to epileptogenesis and excitotoxicity (6). For example, the pro-inflammatory cytokine Interleukin Ib (IL-1b) can interact with the NR2B subunit of the NMDA receptor, resulting in calcium influx across the neuronal membrane and increased excitability (6). A causal role of inflammation in SUDEP is unknown (7). Nejm et al. explored whether fish oil containing the anti-inflammatory n-3 fatty acids DHA and EPA could reduce inflammation in the hearts of rats in a pilocarpine model. Their group found that long-term supplementation with fish oil significantly reduced cardiac levels of IL-6 compared with controls (Nejm et al.). The study did not explore the effect of fish oil on IL-6 levels in the brain, but these findings may provide insight into the mechanism of n-3 fatty acids in reducing seizures (8).

## Summary

This topic adds to current knowledge of the role of genetics, autonomic networks, autonomic physiology and clinical risk factors for SUDEP. It is our hope that this work will encourage basic and clinical scholarship to advance understanding of the mechanisms that underlie SUDEP, and to spur clinical interventions that can prevent SUDEP.

## Author contributions

The author confirms being the sole contributor of this work and approved it for publication.

### Conflict of interest statement

The author declares that the research was conducted in the absence of any commercial or financial relationships that could be construed as a potential conflict of interest.
